# Ramping Position to Aid Non-invasive Ventilation (NIV) in Obese Patients in the ICU

**DOI:** 10.2478/jccm-2023-0002

**Published:** 2023-02-08

**Authors:** Mohamed Shirazy, Christopher Gowers, Padraig Headley

**Affiliations:** 1 Northern Ireland Medical and Dental Training Agency, Belfast, United Kingdom; 2 Ulster Hospital, South Eastern Health and Social Care Trust, Belfast, United Kingdom

**Keywords:** ramping, obese, non-invasive ventilation

## Abstract

**Introduction:**

The ramping position is recommended to facilitate pre-oxygenation and mask ventilation of obese patients in anaesthetics via improving the airway alignment.

**Presentation of case series:**

Two cases of obese patients admitted to the intensive care unit (ICU) with type 2 respiratory failure. Both cases showed obstructive breathing patterns on non-invasive ventilation (NIV) and failed resolution of hypercapnia. Ramping position alleviated the obstructive breathing pattern and hypercapnia was subsequently resolved.

**Conclusion:**

There are no available studies on the rule of the ramping position in aiding NIV in obese patients in the ICU. Accordingly, this case series is significantly important in highlighting the possible benefits of the ramping position for obese patients in settings other than anaesthesia.

## Introduction

Non-invasive ventilation (NIV) is the first-line therapy for treating type 2 respiratory failure in chronic obstructive pulmonary disease (COPD) [1,2]. Despite its efficacy in preventing invasive ventilation, improving gas exchange, and improving survival [3,4], it is susceptible to failure [5]. Applying NIV to obese patients is challenging [6].

Although ramping has been widely studied and recommended for the pre-oxygenation, bag ventilation and intubation of obese patients in anaesthesia [7, 8, 9, 10], there are minimal studies and debates on its rule in intensive care medicine [11, 12, 13, 14]. In this case report, the ramping position has been effectively used to aid the success of NIV in two obese patients with type 2 respiratory failure.

## Presentation of case series

### Case 1

A 72-year-old male patient was admitted to the ICU from the medical ward with type 2 respiratory failure (Table 1) on top of infective exacerbation of COPD and a fractured left neck femur due to a mechanical fall. He had central obesity, BMI of 35.4 kg/m^2^, hight of 171cm, weight 103.5 kg, and neck circumference of 47 cm. He had a background history of hypertension, COPD, heavy smoking and alcohol dependence. He was managed in the ward with NIV using the BiPAP machine (NIPPY 3+). A few hours after commencing NIV, his respiratory acidosis significantly deteriorated, and he became drowsy.

**Table 1:** ABG, Blood results, and ventilation of Case 1

	Admission	After ramping	12 hrs	24 hrs	Day 3	Day 5
ABG^*^						
PH	7.15	7.28	7.46	7.43	7.42	7.41
PCO_2_** (Kpa)	16.9	10.9	7	7.1	7.5	7.4
PO_2_ ***(Kpa)	9.5	9.7	9.2	8.5	8.9	8.3
HCO_3_****	30.8	31.3	33	31.8	32.2	32.4
(mmol/l)						
Lactate (mmol/l)	1.1	0.9	1.3	1.5	0.8	1.1

**Ventilation**						
Oxygen delivery	NIV mask Drager Evita V800	NIV mask Drager Evita V800	NIV mask Drager Evita V800	Nasal cannula	Nasal cannula	Room air
Ventilator mood	BIPAP	BIPAP	CPAP			
Airway adjunct	Naso-pharyngeal	Naso-pharyngeal	Naso-pharyngeal	Natural	Natural	Natural
FIO_2_*****	40%	40%	30%	36%	32%	21%
PEEP****** (cmH_2_O)	8	8	8			
Pressure support (cmH_2_O)	23	23	N/A			
Actual tidal volume (ml)	125	428	538			
Peek inspiratory pressure (cmH_2_O)	42	24	23			
Respiratory rate	42	28	22	20	18	17
WOB scale	6/7	4/7	2/7	1/7	1/7	1/7

Blood results						
Haemoglobin (g/l)	172			165	170	168
leukocytes (x10 g/l)	13.2			11	7.8	8
Platelets (x10 g/l)	169			195	209	218
C-Reactive Protien (mg/l)	54			22.6	17.5	11.4

*Arterial blood gas; **Partial pressure of carbon dioxide; ***Partial pressure of oxygen; ****Bicarbonate; *****Fraction of inspired oxygen; ******Positive end expiratory pressure

**Fig. 1:**
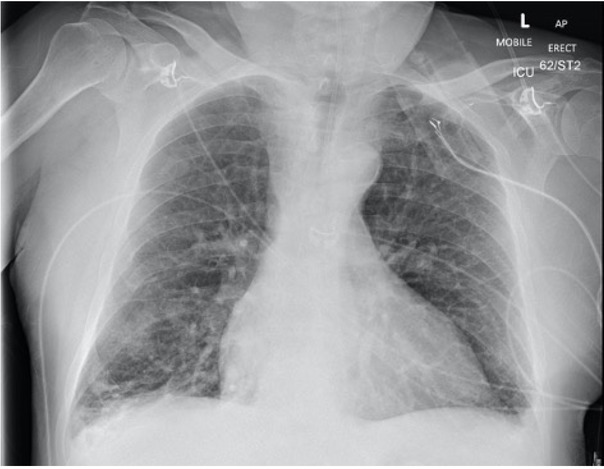
Chest X-ray of Case 1: Bi-basal atelectasis and small bilateral pleural effusion.

In the ICU, NIV using the ventilator (Drager Evita V800) was applied, which showed an obstructive waveform. Clinical examination and a chest X-ray ruled out bronchospasm and pneumothorax (Figure 1). The obstructive breathing pattern improved partially following the insertion of a nasopharyngeal airway. The patient was positioned in the ramping position using an Oxford HELP pillow, ultimately and immediately resolving the obstructive breathing pattern. Subsequently, respiratory acidosis resolved. He was successfully weaned from NIV 24 hours later (Table 1). Additionally, he was treated with broad spectrum antibiotics, steroids & nebulisers (Table 3).

On his third ICU Day, he got a hemiarthroplasty under spinal anaesthesia and a femoral nerve block, and got weaned from nasal oxygen.

### Case 2

**Fig. 2:**
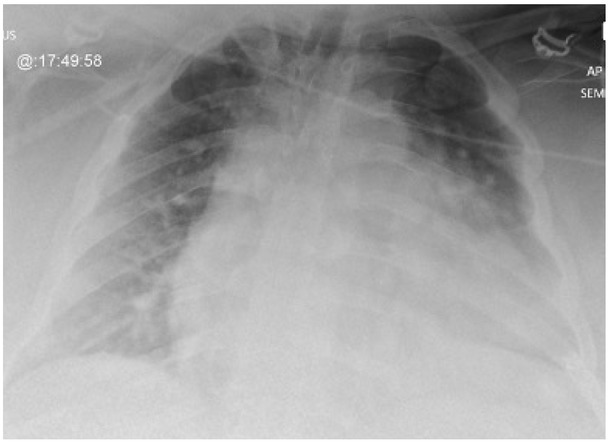
Chest X-ray of Case 2: Hazy opacification of both lungs’ mid and lower zones. Infection versus congestion.

A 37-year-old male patient was admitted to the ICU from the emergency department (ED) with severe type 2 respiratory failure (Table 2) due to community acquired pneumonia. He had hypogonadism and morbid obesity, BMI of 64kg/m^2^, hight of 169 cm, weight of 183 kg, and neck circumference of 56 cm. Moreover, he smoked shisha and had recovered of COVID-19 infection three months earlier. His blood results showed raised inflammatory markers (Table 2), and his chest X-ray showed pulmonary congestion and infiltrates (Figure 2).

**Table 2:** ABG, Blood results, and ventilation of Case 2

	Admission	After ramping	12 hrs	24 hrs overnight	Day 3	Day5
ABG*						
PH	7.17	7.22	7.43	7.46	7.44	7.43
PCO_2_** (Kpa)	15.3	13.2	8.6	8.6	8	7.6
PO_2_*** (Kpa)	26.8	10.9	14.9	9.5	8.9	11.7
HCO_3_**** (mmol/l)	31.5	31.7	36.6	39	35.5	33.6
Lactate (mmol/l)	0.8	0.8	1.3	0.8	1.2	1.1

**Ventilation**						
Oxygen delivery	NIV mask Drager Evita V800	NIV mask Drager Evita V800	Face mask	NIV mask Drager Evita V800	Nasal cannula	Room air
Ventilator mood	BIPAP	BIPAP		BIPAP		
Airway adjunct	Natural	Natural	Natural	Natural	Natural	Natural
FIO_2_*****	100 %	60 %	40%	40 %	24 %	21%
PEEP****** (cmH_2_O)	5	5		7		
Pressure support (cmH_2_O)	25	25		14		
Actual tidal volume (ml)	170	507		480		
Peek inspiratory pressure (cmH_2_O)	39	26		27		
Respiratory rate	38	29	22	18	20	22
WOB scale	6/7	5/7	2/7	1/7	1/7	2/7

**Blood results**						
Haemoglobin (g/l)	118			106	112	116
Leukocytes (x10 g/l)	15.9			9	9.8	10.2
Platelets (x10 g/l)	368			302	299	296
C-Reactive Protein (mg/l)	76.2			52.8	26.7	26.2

*Arterial blood gas; **Partial pressure of carbon dioxide; ***Partial pressure of oxygen; ****Bicarbonate; *****Fraction of inspired oxygen; ******Positive end expiratory pressure

**Table 3:** Medications for Case 1.

Medication	Dose and route	Duration
-Co-amoxiclav	1.2 g Intravenous twice daily	3 days
-Clarithromycin	500 mg Intravenous twice daily	3 days
-Doxycycline	100 mg Oral twice daily	4 Days
-Pantoprazole	40 mg Intravenous once daily	5 days
-Prednisolone	30 mg Oral once daily	5 days
-Enoxaparin	40 mg Subcutaneous once daily	5 days
-Amlodipine	5 mg Oral once daily	Regular medication
-Paracetamol	1g Intravenous every 6 hours	5 days
-Oxycodone	2.5 mg Oral every 4 hours	5 days as per request
-Salbutamol	5 mg nebuliser every 6 hours	5 days
-Ipratropium Bromide	500 mcg nebuliser every 6 hours	5 days

**Fig.-3:**
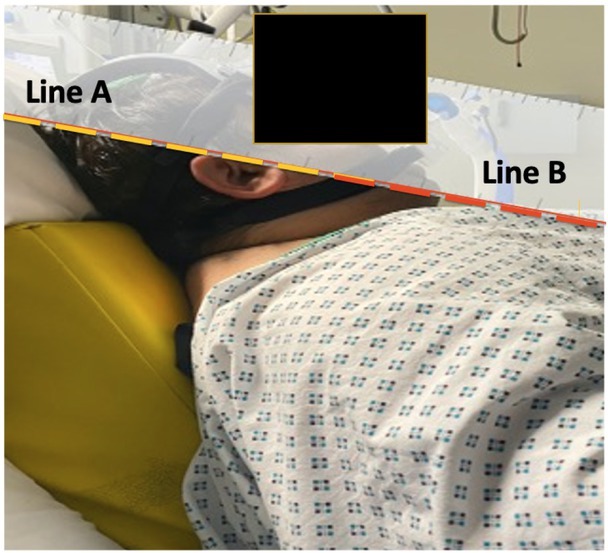
Case 2 on NIV lying on Oxford HELP pillow. The external auditory meatus (Line A) and the sternal notch (Line B) are aligned.

The NIV trial in the ED failed to improve his hypercapnia. In the ICU, NIV was associated with an obstructive breathing pattern, low tidal volume, and high peek pressures seen on the ventilator monitor (Drager Evita V800). An immediate resolution of the obstructive breathing pattern was achieved once he was placed in a ramping position using an Oxford HELP pillow (Figure 3).

The arterial blood gas showed marked resolution of the hypercapnia and acidosis (Table 2). He received broad-spectrum antibiotics and diuretics (Table 4). Twelve hours later, he was weaned from NIV and placed on humidified oxygen during the day and NIV at night.

**Table 4:** Medications for Case 2.

Medication	Dose and route	Duration
-Co-amoxiclav	1.2 g Intravenous twice daily	5 days
-Clarithromycin	500 mg Intravenous twice daily	5days
-Pantoprazole	40 mg Intravenous once daily	5 days
-Prednisolone	30 mg Oral once daily	5 days
-Enoxaparin	80 mg Subcutaneous once daily	5 days
-Furosemide	40 mg Intravenous twice daily	4 days
-Salbutamol	5 mg nebuliser every 6 hours	5 days
-Ipratropium Bromide	500 mcg nebuliser every 6 hours	5 days

Oesophageal manometry was not used in both cases to assess the work of breathing (WOB) due to its impracticality during the acute phase in non-intubated patients; however, a WOB scale from 1-7 was used instead (Table 1, Table 2).

In both cases, oral feeding was started within the first 24 hours. They received isocaloric high protein diet based on their adjusted body weight.

Both patients were transferred to the respiratory ward after their fifth ICU Day for further pulmonary function tests and sleep studies. Afterwards, they were discharged home.

Case 1 was given a follow-up appointment with the orthogeriatric nurse, dietitian, and the smoke cessation team. Case 2 was given a home BiPAP machine and a dietitian appointment.

## Discussion

The prevalence of obesity in ICU is around 20% [15]. Obesity is associated with difficult mask ventilation due to excess pharyngeal fat, macroglossia, and small mouth opening [16]. Also, increased neck circumference > 42 cm is an independent risk factor for difficult mask ventilation [17]. In both cases, the obstructive breathing pattern on the NIV was attributed to the patients’ high body mass index (BMI), which was 35.4 kg/ m2 and 64 kg/m2, respectively, and large neck circumference, 47 cm and 56 cm, respectively.

Obesity is associated with significant increase in respiratory resistance, airway narrowing and closure, and airway hyperresponsiveness. Fat deposition in the abdomen and mediastinum increases the chest wall stiffness resulting in reduced compliance of the lungs and the whole respiratory system and alters the breathing pattern. Accordingly, lung expiratory reserve volume (ERV), functional residual capacity (FRC), and tidal volume are reduced resulting in a shallow breathing pattern. These alterations are compensated for via increased respiratory rate and increased minute ventilation accordingly. Besides, obesity slightly reduces the forced vital capacity (FVC) and the forced expiration in the first second (FEV1) [18]. Jones and Nzekwu. (2006) concluded that the reduction in the FRC is directly proportionate to the severity of obesity; however, residual volume (RV) and total lung capacity (TLC) are preserved even in severe obesity. Additionally, gas trapping indicated by the RV-to-TLC ratio remained normal or minimally increased [19]. Central obesity & high BMI > 62 kg/m2 are associated with reduced FEV1/FVC ratio leading to obstructive and restrictive breathing pattern [20,21].

NIV entails applying a mask or similar device to the face to deliver supported ventilation. It has been increasingly used to treat respiratory failure and prevent invasive ventilation over the past two decades [1, 2, 3, 4]. Obese patients require more extended time and militant ventilator settings to achieve adequate resolution of hypercapnia [6]. Apigo et al. (2020) developed a useful WOB scale to monitor the need for intubation in COVID pneumonia patients. The scale is from 1-7 and is based on the respiratory rate and the use of accessory muscles [22].

On inducing anaesthesia, obese patients lose their neuromuscular control of the upper airway. The tongue falls by gravity towards the posterior pharynx, resulting in obstruction and difficult mask ventilation [23]. Similarly, in severe hypercapnia, patients lose the airway anatomical balance, making administering NIV difficult. Anaesthetists overcome this anatomical imbalance by opening the airway via jaw thrust, using airway adjuncts such as oropharyngeal and nasopharyngeal airways, placing the head in a sniffing position, and elevating the head [23,24].

**Fig. 4:**
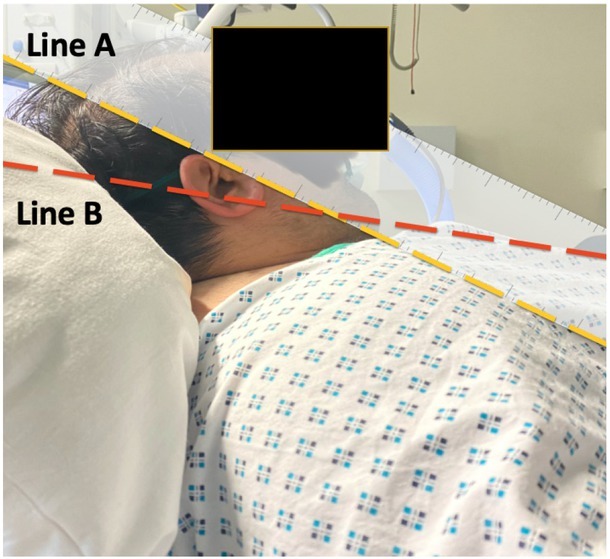
Case 2 lying at 30 degrees up of the thorax. The external auditory meatus (line A) and the sternal notch (Line B) are not aligned.

Ramping elevates the upper body, head at 25°, and neck to achieve horizontal alignment of the external auditory meatus and the sternal notch [25]. This position can be achieved by placing blankets or using an elevation pillow under the upper back and shoulders [10, 25]. This position is different than breaking the ICU bed and elevating it 30° up the thorax which does not align the external auditory meatus and the sternal notch (Figure 4).

Unlike all other published studies on the usefulness of ramping in airway management in anaesthesia, Semler et al. (2017) concluded that ramping did not improve pre-oxygenation and increased the number of intubation-attempts in the ICU [11].

No available studies exist on the effectiveness of ramping during NIV. The same concept of ramping in anaesthesia was implemented in the cases as mentioned earlier to alleviate the obstructive airway component induced by obesity via adequate airway alignment (Figure 3). The immediate alleviation of the obstructive breathing pattern after ramping, in both cases, is a positive indicator towards its efficacy and the need for further studies.

## Conclusions

NIV is an effective therapy for type-2 respiratory failure but can be challenging in obese patients.

The ramping position can be a valuable adjunct to aid NIV in obese patients.

Further studies are required on the effectiveness of ramping positions during NIV in the ICU.
